# Wide-field endoscope accessory for multiplexed fluorescence imaging

**DOI:** 10.1038/s41598-023-45955-x

**Published:** 2023-11-09

**Authors:** Gaoming Li, Miki Lee, Tse-Shao Chang, Joonyoung Yu, Haijun Li, Xiyu Duan, Xiaoli Wu, Sangeeta Jaiswal, Shuo Feng, Kenn R. Oldham, Thomas D. Wang

**Affiliations:** 1 Division of Gastroenterology, Department of Internal Medicine, University of Michigan, 109 Zina Pitcher Pl. BSRB 1522, Ann Arbor, MI 48109-2200 USA; 2 Department of Mechanical Engineering, University of Michigan, Ann Arbor, MI 48109 USA; 3 Department of Biomedical Engineering, University of Michigan, Ann Arbor, MI 48109 USA

**Keywords:** Cancer imaging, Colonoscopy, Biomedical engineering, Electrical and electronic engineering, Mechanical engineering, Imaging and sensing, Wide-field fluorescence microscopy

## Abstract

A wide-field endoscope that is sensitive to fluorescence can be used as an adjunct to conventional white light endoscopy by detecting multiple molecular targets concurrently. We aim to demonstrate a flexible fiber-coupled accessory that can pass forward through the instrument channel of standard medical endoscopes for clinical use to collect fluorescence images. A miniature scan mirror with reflector dimensions of 1.30 × 0.45  mm^2^ was designed, fabricated, and placed distal to collimated excitation beams at λ_ex_ = 488, 660, and 785 nm. The mirror was driven at resonance for wide angular deflections in the X and Y-axes. A large image field-of-view (FOV) was generated in real time. The optomechanical components were packaged in a rigid distal tip with dimensions of 2.6 mm diameter and 12 mm length. The scan mirror was driven at 27.6 and 9.04 kHz in the fast (X) and slow (Y) axes, respectively, using a square wave with 50% duty cycle at 60 V_pp_ to collect fluorescence images at 10 frames per sec. Maximum total divergence angles of ± 27.4° and ± 22.8° were generated to achieve a FOV of 10.4 and 8.4 mm, respectively, at a working distance of 10 mm. Multiplexed fluorescence images were collected in vivo from the rectum of live mice using 3 fluorescently-labeled peptides that bind to unique cell surface targets. The fluorescence images collected were separated into 3 channels. Target-to-background ratios of 2.6, 3.1, and 3.9 were measured. This instrument demonstrates potential for broad clinical use to detect heterogeneous diseases in hollow organs.

## Introduction

Improved methods are needed to detect diseases that originate from the mucosal surface of hollow organs, such as colon^1–3^. Endoscopes can be inserted into natural body orifices to access this highly metabolically active layer of tissue that serves as the origin of many human disorders^4–6^. Images can be collected in vivo in real time with high spatial resolution to identify and localize abnormal regions. White light illumination is currently used to identify pre-malignant masses, such as polyps, but has limited effectiveness to identify lesions that are flat and subtle in appearance^7–9^. Fluorescence can be used to label ligands, such as peptides, and provide high contrast to distinguish regions of pathology from surrounding areas of normal tissues. The optical spectrum provides a wide range of wavelengths (500–900 nm) that can be separated into different channels used to perform multiplexed detection of unique molecular targets^10–12^. A number of fluorophores, such as fluorescein and indocyanine green (ICG), are FDA-approved for clinical administration, and can be used to label ligands that bind specifically to biomarkers of disease^13^. Medical endoscopes have working channels that are typically several millimeters in diameter, and can pass instruments for tissue resection^4–6^. A flexible, fiber-coupled accessory can pass through this channel if the dimensions are sufficiently small.

The rigid distal tip of conventional medical endoscopes contains a complex arrangement of objective lenses to achieve high spatial resolution with a large image field-of-view (FOV)^6^. Barrel distortion is introduced by increasing the magnification in the center versus that in the periphery to achieve a maximum divergence angle up to 140° (± 70°) or more. Achromats are used to correct for chromatic dispersion so that light over the visible spectrum (400–700 nm) can focus on a detector. The pixel number in the sensor is typically much greater than the number of fibers in a flexible optical bundle. Alternatively, images can be generated over a large FOV using a much simpler strategy by deflecting a low numerical aperture (NA) collimated beam at wide angles. A miniature scan mirror placed distal to the collimating optics can deflect a diffraction-limited beam over an arbitrarily large FOV. The excitation beam passes through the center of the objective where sensitivity to aberrations is minimal. In this configuration, the FOV is limited only by the mechanical scan angle of the reflector. The instrument can be scaled down in diameter without loss of resolution and fiber-coupled for use as an accessory with medical endoscopes. Simple fabrication processes can be developed with potential for mass manufacture at low cost.

Micro-Electro-Mechanical Systems (MEMS) methods were used to design and fabricate miniature scan mirrors for use in the distal end of flexible fiber-coupled instruments^14–16^. Small dimensions allow for placement in the post-objective position, and allows for the distal tip to be scaled down in size. These devices were batch fabricated using simple processes on a single silicon wafer for mass manufacture^17^. By comparison, conventional endoscopes use bulky optics that are large in dimension and require multiple focusing elements. Here, we aimed to scale down the dimensions of a wide-field accessory to < 2.6 mm diameter and < 12 mm length. This small size allowed for forward passage through the instrument channel of standard medical endoscopes. A side-view geometry provides direct beam delivery to the mucosal surface without requiring bending of the distal tip. Multiplexed fluorescence images were collected using excitation at λ_ex_ = 488, 660, and 785 nm to demonstrate use of a broad range of wavelengths in the visible and NIR spectrum. In vivo images were collected from adenomas that arise spontaneously in a pre-clinical model of colorectal cancer. Disease heterogeneity was addressed by detecting 3 molecular targets concurrently.

## Results

### Imaging system

**Figure 1:**
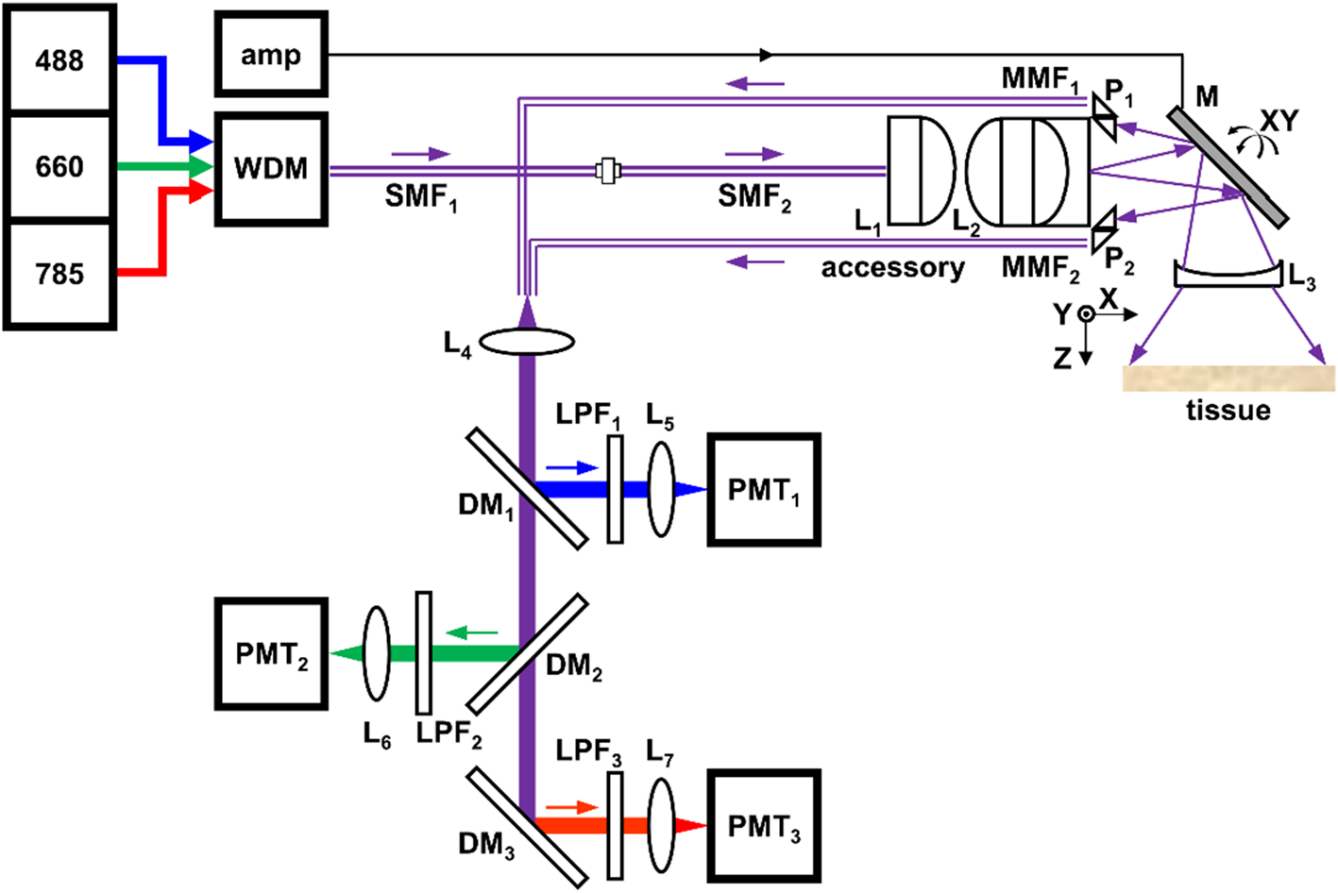
Imaging system. Schematic is shown for multiplexed detection of fluorescence images using λ_ex_ = 488, 660, and 785 nm by the wide-field accessory. Details are provided in text. Key: *amp* amplifier, *DM* dichroic mirror, *L* lens, *LPF* low-pass filter, *M* mirror, *MMF* multi-mode fiber, *P* prism, *PMT* photomultiplier, *SMF* single-mode fiber, *WDM* wavelength division multiplexer.

A schematic is shown of the imaging system used to support the wide-field accessory to collect fluorescence images in 3 channels, Fig. 1. The excitation beams were combined using a custom wavelength division multiplexer (WDM) for delivery through a single mode fiber (SMF_1_). A second single-mode fiber (SMF_2_) was centered in the wide-field accessory using a ferrule. The accessory was coupled to the excitation fiber SMF_1_ using an FC/APC connector. The excitation beams exciting SMF_2_ were collimated using an aspheric (L_1_) and achromatic (L_2_) lens arranged in tandem. A miniature mirror (M) deflected the beams at a 90° angle, and performed wide angular scanning about the X and Y axes. A concave lens (L_3_) provided additional angular divergence, and served as a window to protect the accessory components. Laser power at λ_ex_ = 488, 660, and 785 nm was transmitted out of the accessory with 22%, 16%, and 9% efficiency, respectively. During imaging, laser power between 3 and 5 mW was incident on the tissue. Visible and NIR fluorescence was collected by L_3_, and descanned by M. Prisms P_1_ and P_2_ with dimensions of 0.4 mm × 0.3 mm (hypotenuse × legs) were used to increase the effective area for fluorescence collection and delivery into 2 multi-mode fibers (MMF_1_ and MMF_2_). Lens L_4_ collimated the fluorescence beams for separation into 3 spectrally distinct channels using a set of dichroic mirrors DM_1_-DM_3_, long pass filters LPF_1_-LPF_3_, and lenses L_5_-L_7_ for detection by photomultiplier tubes PMT_1_-PMT_3_.

### Optical design of wide-field accessory

**Figure 2:**
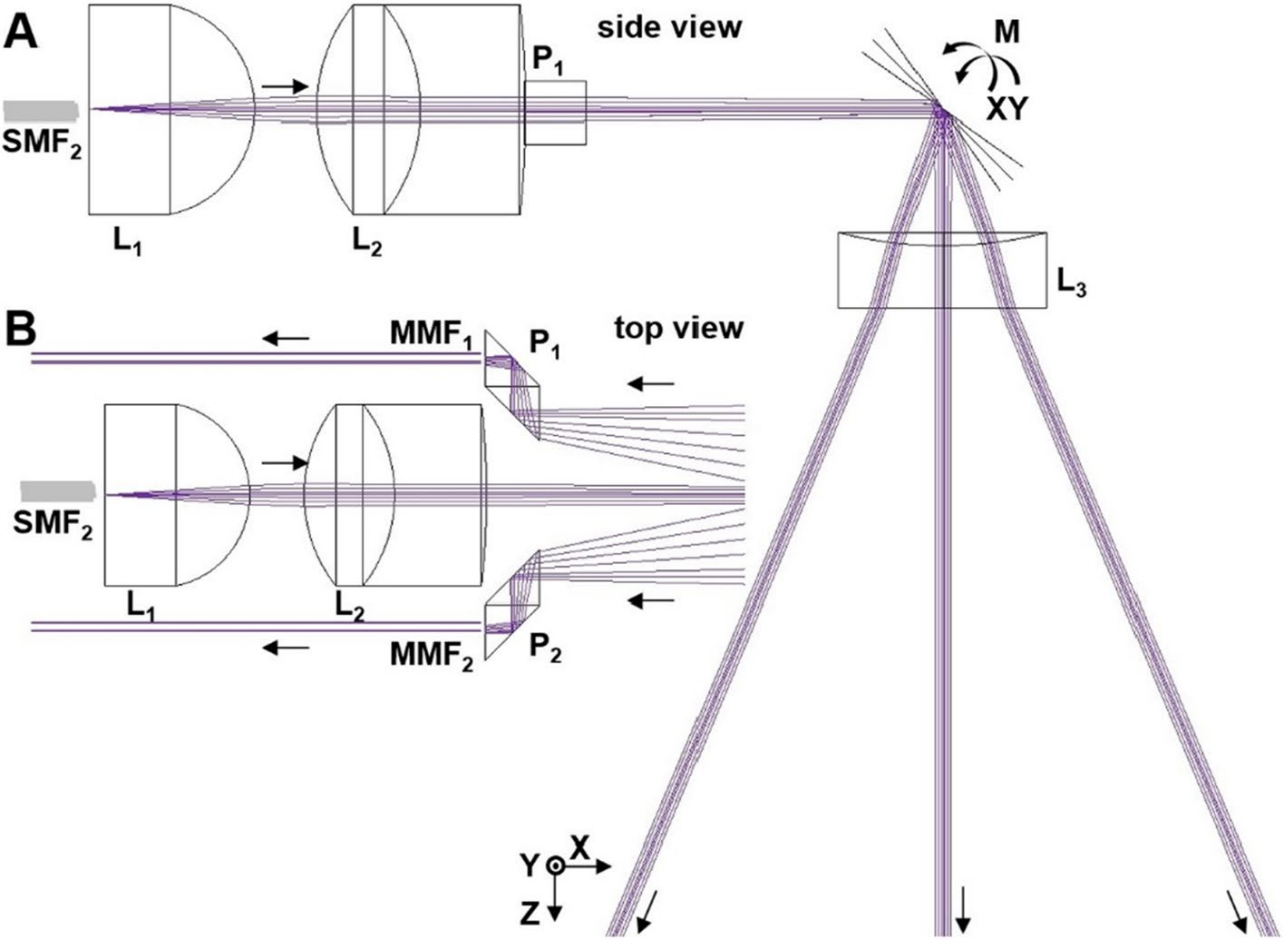
Optical design. Ray trace simulations show the excitation and emission beam paths. (**a**) An aspheric and achromat lens collimate the excitation beam exiting SMF_2_ with minimal chromatic dispersion (side view). A miniature scan mirror (M) deflects the collimated beam at wide angles. A concave lens further increases the deflection angles. (**b**) Fluorescence is collected over a broad spectral bandwidth (500–900 nm) using a pair of prisms that are coupled to multi-mode fibers (MMF) located along either side of the accessory (top view).

A simple optical design was used to deliver a diffraction-limited collimated beam for rapid deflection at wide angles in the X and Y axes by mirror M to achieve a large image FOV, Fig. 2. Ray trace simulations were performed to determine the FOV over a range of working distances WD = 0 to 50 mm. Commercially available lenses were used, including an aspheric (L_1_, 1.0 mm diameter, f = 0.60 mm, NA = 0.83, n = 1.84), achromatic doublet (L_2_, 1.0 mm diameter, f = 1.5 mm, NA = 0.33, n = 1.62 and 1.84), and concave (L_3_, 1.0 mm diameter, f = − 4 mm, n = 1.48). The optimal center-to-center distance between L_1_ and L_2_ to collimate the excitation beams was 1.19 mm. The optics were chosen to fit within the physical constraints of the accessory that could pass through the working channel of a standard medical endoscope. The resulting NA was 0.00015, and the beam diameter was 28.9 µm at WD = 10 mm. With the addition of L_3_, the maximum optical scan angles increased from ± 27.30° and ± 28.03° to ± 32.75° and ± 33.40° in the X and Y axes, respectively. This optical design allowed for a broad range of fluorescence wavelengths to be collected over the visible and NIR spectrum (λ_em_ = 500–900 nm). Multiplexed imaging could be performed with minimal chromatic aberrations over a large image FOV. In the simulations, a FOV_x_ and FOV_y_ of 11.25 and 9.12 mm were achieved at WD = 10 mm, Table S1. Ray trace simulations were performed to characterize chromatic aberrations at 488, 660, and 785 nm, Table S2, resolution at different positions in the image FOV, Table S3, and collection efficiency as a function of scan angle, Fig. S3.

### Scan mirror

**Figure 3:**
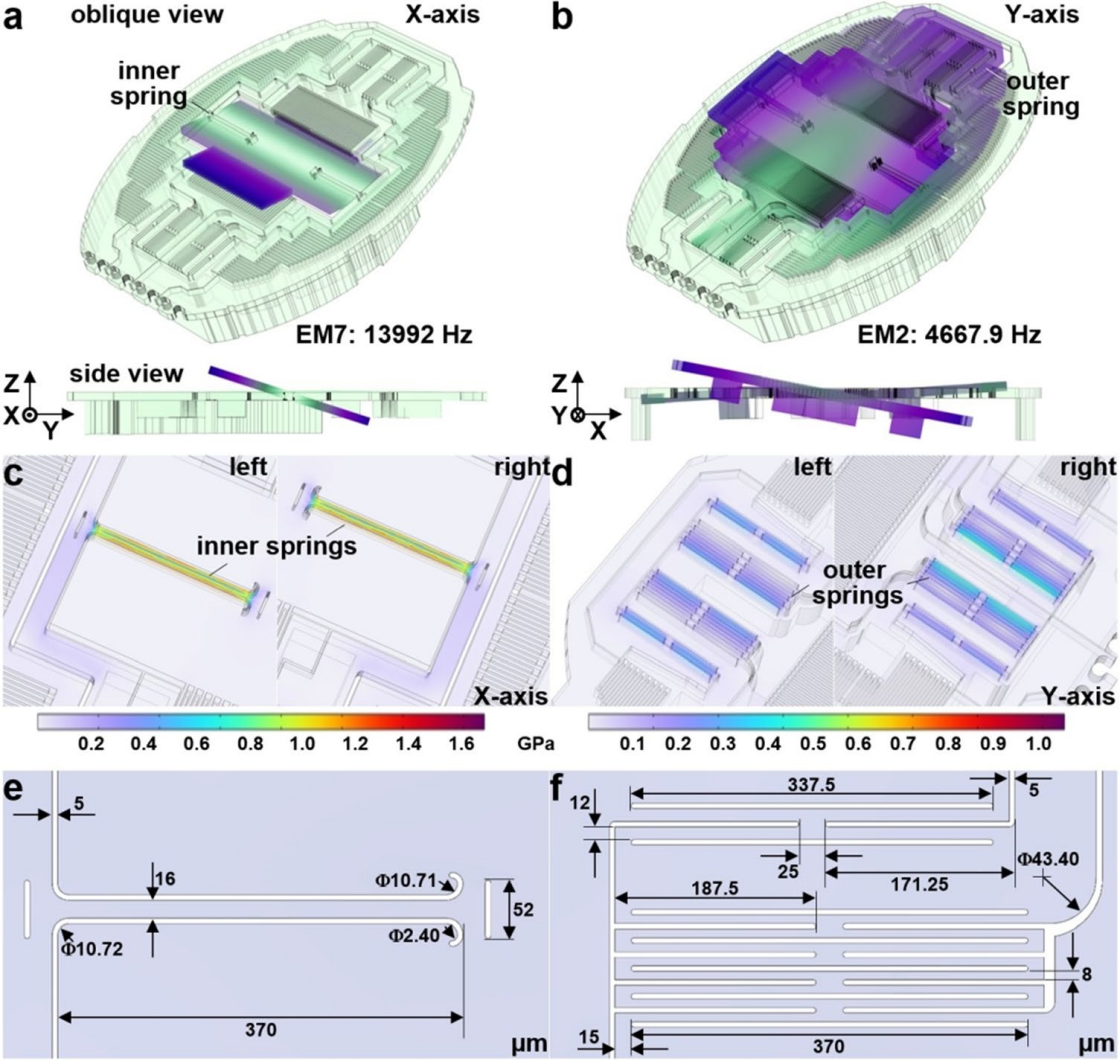
Spring design. CAD drawing shows areas of simulated active scan mirror tilt motion in the (**a**) X and (**b**) Y-axes at eigenmodes EM_7_ = 13,992 and EM_2_ = 4667.9 Hz, respectively (oblique view). Angular deflections of scan mirror and support from the backside islands are shown (side view). The color map shows the total displacement magnitude (pale green to dark blue corresponds to zero to max amplitude). Mechanical stress is shown for the (**c**) 2 inner torsional springs arranged in-line along the reflector length, and for (**d**) symmetric sets of 4 outer torsional springs. The maximum stresses applied to the springs are 1.69 and 1.07 GPa for the inner and outer springs, respectively, which are lower than the tensile strength of silicon. The dimensions of the inner (**e**) and outer (**f**) torsional springs are shown.

A compact 2D scan mirror (M) was designed with dimensions to fit in a distal rigid tip of a wide-field accessory with 2.6 mm diameter and 12 mm length. A reflector with dimensions of 1.30 × 0.45 mm was mounted on a 2.02 × 2.5 mm gimbal to minimize cross-talk between the X and Y axes. Finite element analysis was performed using COMSOL software. Eigenmodes EM7 = 13,992 Hz and EM2 = 4667.9 Hz, respectively, were identified for maximum X and Y axes tilt motions, Fig. 3a,b. The resonance frequency in the X-axis was determined by 2 inner torsional springs arranged in-line along the reflector length, Fig. 3c. Regions of increased stress are shown in pseudo-color. The resonance frequency in the Y-axis was determined by 4 outer torsional springs arranged in 2 symmetric sets on either side of the reflector, Fig. 3d. The dimensions of the springs are shown, Fig. 3e,f.

**Figure 4:**
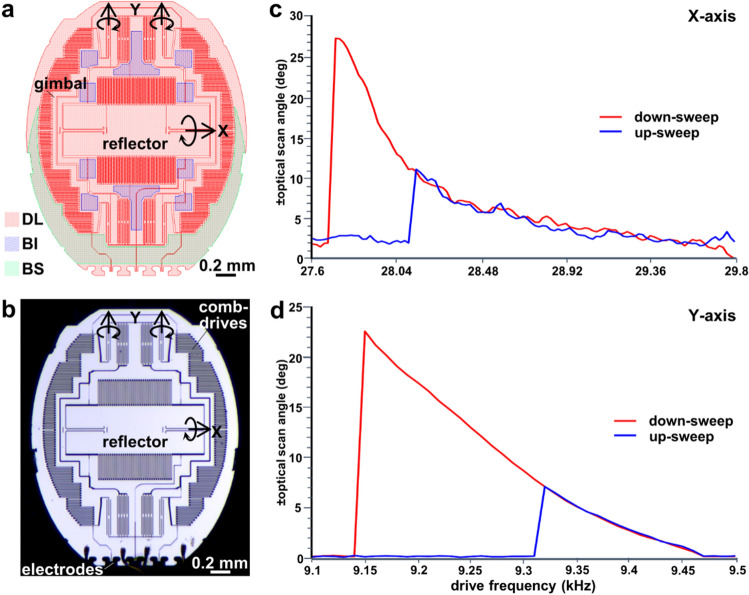
Scan mirror design. (**a**) A CAD layout of the 2D scan mirror (M) is shown. The locations of the device layer (DL), backside islands (BI), and backside substrate (BS) are identified. A reflector with dimensions of 1.30 × 0.45 mm is mounted on a 2.02 × 2.5 mm gimbal that rotates about the X and Y axes to deflect the beams over wide angles. (**b**) Photo is shown of a fabricated scan mirror. (**c**,**d**) The frequency response of the scan mirror is shown using down-sweep (high-to-low frequencies) and up-sweep (low-to-high frequencies). Maximum optical scan angles of ± 27.4° and ± 22.8° were achieved in the X and Y axes using a square wave drive signal at 27.75 and 9.15 kHz, respectively, with 50% duty cycle at 60 V_pp_ during down-sweep of frequencies.

A CAD drawing is shown of the front-view of the scan mirror, Fig. 4a. A photo of the fabricated device is shown, Fig. 4b. The frequency response in the X and Y axes was generated using a down-sweep (high-to-low frequencies) and up-sweep (low-to-high frequencies), Fig. 4c,d. A time interval of ~ 1 s was employed in between frequency transitions to provide stable scan mirror motions at each drive frequency and to maximize the scan angles at resonance. Optical scan angles of ± 27.4° and ± 22.8° were measured in the X and Y axes, respectively. The drive frequencies measured *2ω* agreed with the simulation results for *ω*.

### Packaging of wide-field accessory

**Figure 5:**
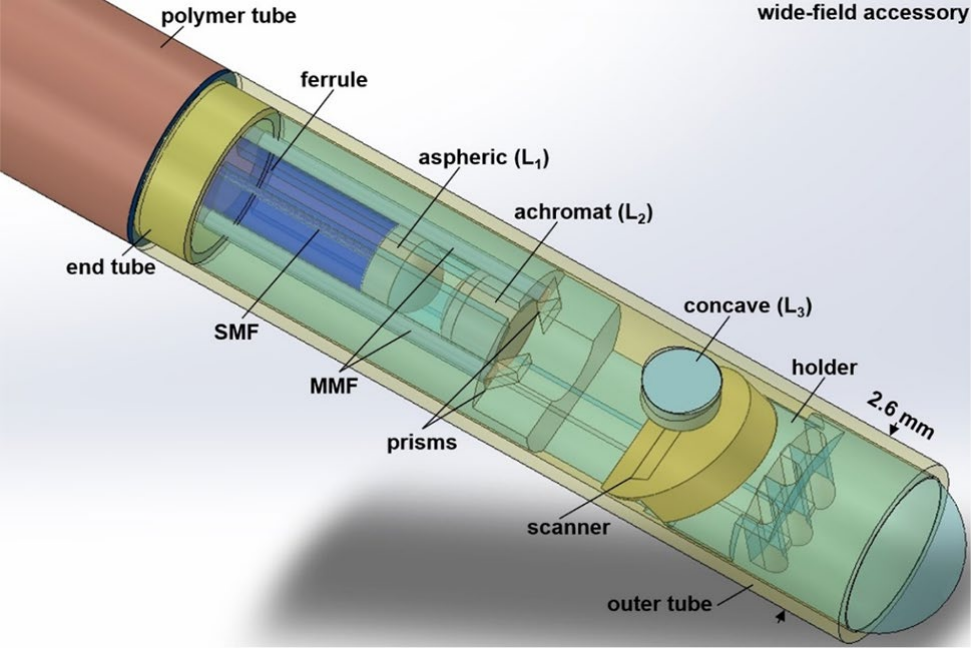
Packaging strategy. CAD drawing shows the arrangement of individual optical and mechanical components in the rigid distal tip of the wide-field accessory.

**Figure 6:**
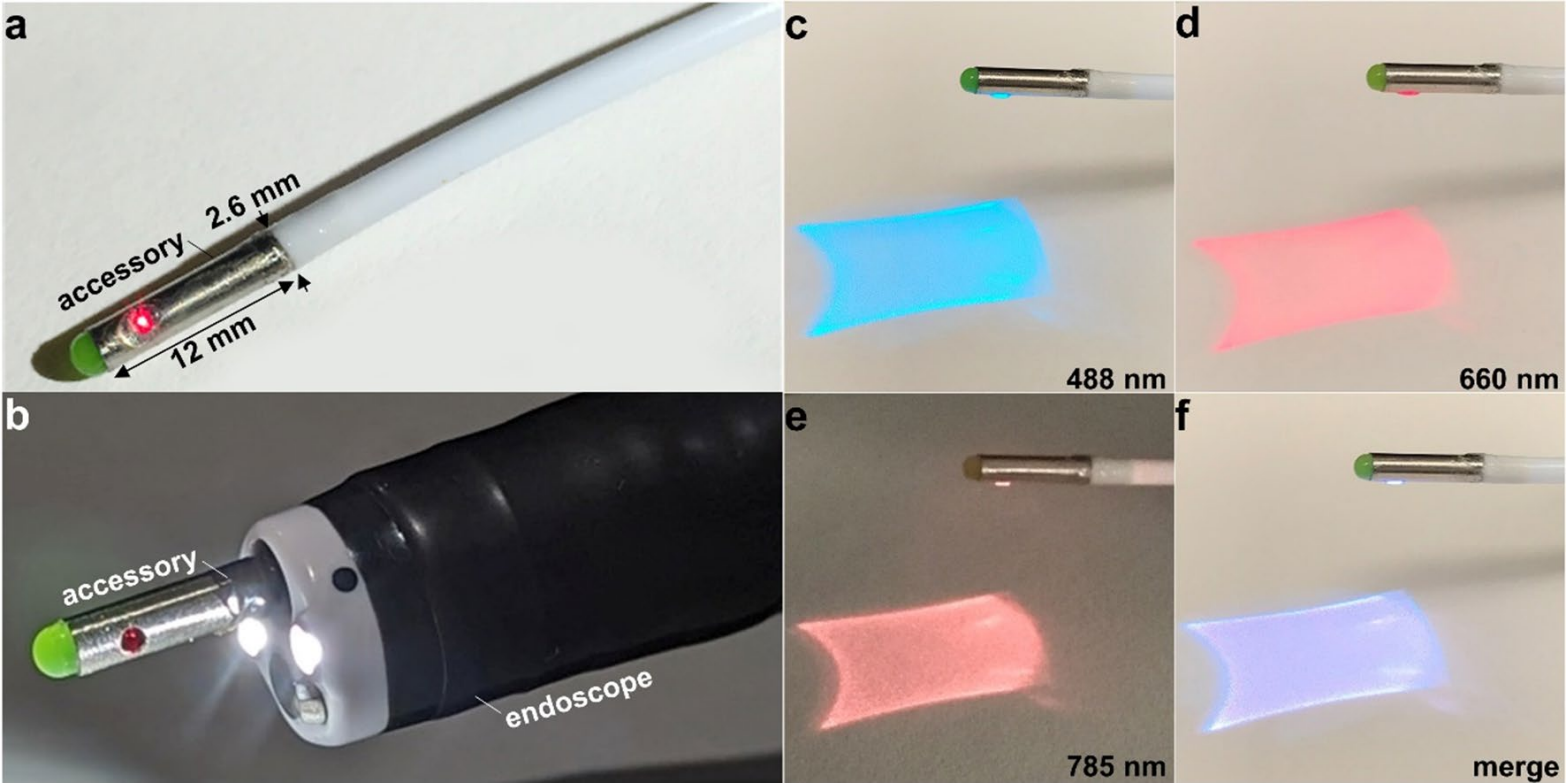
Endoscope accessory. (**a**) The rigid distal tip of the flexible fiber-coupled wide-field accessory has dimensions of 2.6 mm diameter and 12 mm length. (**b**) The accessory passes forward easily through the 3.7 mm diameter instrument channel of a standard medical endoscope (Olympus, #GIF-1TH190). Photos show the scan mirror deflecting the excitation beams in a Lissajous pattern for λ_ex_ = (**c**) 488, (**d**) 660, and (**e**) 785 nm at a working distance WD = 20 mm. (**f**) Concurrent excitation of 3 wavelengths is shown in the merged image.

The wide-field accessory was packaged in a stainless-steel outer tube with a 2.6 mm OD, 2.3 mm ID, and 12 mm length. A CAD drawing of the rigid distal tip shows the spatial arrangement of the internal components, Fig. 5. A photo of the flexible fiber-coupled wide-field accessory is shown after completion of packaging, Fig. 6a. This instrument passed forward seamlessly through the working channel of a standard medical endoscope, Fig. 6b. Delivery of excitation at λ_ex_ = 488, 660, and 785 nm separately, Fig. 6c–e, and concurrently, Fig. 6f, shows a large image FOV.

### Image characterization

The optical scan angle of the miniature mirror was confirmed by collecting fluorescence images from a grid target consisting of black dots spaced 1 mm apart at a working distance WD = 10 mm. The target was stained with fluorescence dyes, including FITC, Cy5.5, and IRDye800, for excitation with λ_ex_ = 488, 660, and 785 nm, respectively. Fluorescence images were collected using the 3 excitation beams separately, Fig. S1a–c, and all together, Fig. S1d. A measured FOV of 10.4 × 8.4 mm^2^ was supported by detection of 10 and 9 black dots in the X and Y directions, respectively. Lateral resolution was characterized by measuring the full-width-at-half-maximum (FWHM) of the point spread function (PSF) in the X and Y axes at WD ranging between 0 and 50 mm, Fig. S2a,b. A FWHM of 307.7 µm, 198.8 µm, and 185.6 µm and 159.1 µm, 168.2 µm, and 168.3 µm was measured at WD = 10 mm in the X and Y axes, respectively, for λ_ex_ = 488, 660, and 785 nm, respectively.

### Multiplexed wide-field imaging in vivo

**Figure 7:**
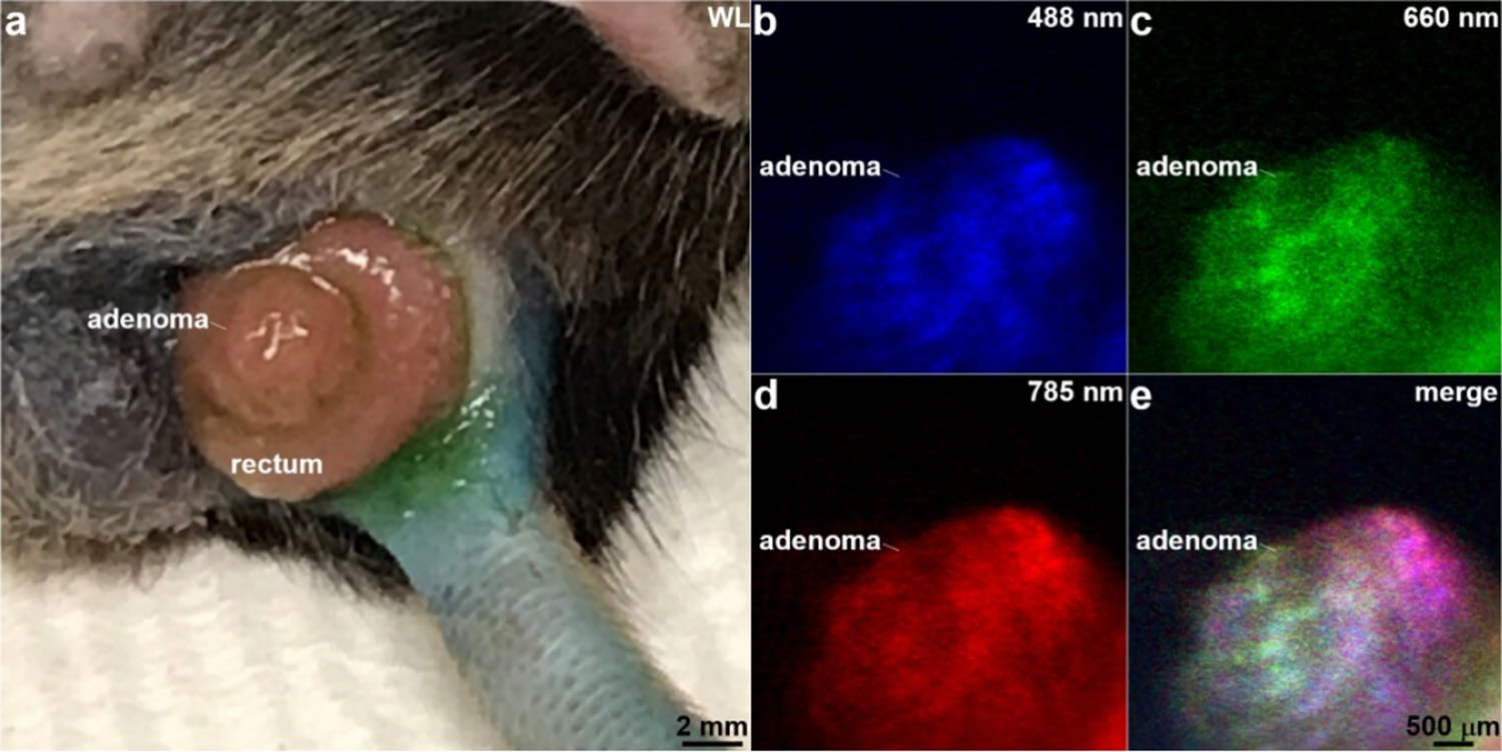
In vivo imaging. (**a**) Photo is shown of an ~ 4 mm diameter adenoma located adjacent to normal mucosa in the prolapsed rectum of a live *CPC;Apc* mouse. Fluorescence images were collected at a working distance of WD = 10 mm using excitation λ_ex_ = (**b**) 488, (**c**) 660, and (**d**) 785 nm to detect fluorescently-labeled peptides KCC*-FITC, QRH*-Cy5, and KSP*-IRDye800, specific for Prdx1, EGFR, and ErbB2, respectively. (Supplementary Videos 1–3) Target-to-background (T/B) ratios of 2.6, 3.1, and 3.9, respectively, were measured. (**e**) The merged image shows use of all 3 excitation wavelengths concurrently. Images were captured at 10 fps. (Supplementary Video 4).

Multiplexed fluorescence images were collected in vivo from *CPC;Apc* mice that have been genetically engineered to spontaneously develop colonic adenomas, Fig. 7a. Fluorescently-labeled peptides, including KCC*-FITC, QRH*-Cy5, and KSP*-IRDye800, Fig. S4a–c, were topically administered to a region of prolapsed rectum. The fluorophores were chosen with absorption spectra that match the laser excitation wavelengths at λ_ex_ = 488, 660, and 785 nm with minimal overlap of fluorescence emission spectra, Fig. S4d,e. The peptide ligands bind specifically to Prdx1, EGFR, and ErbB2, respectively. These molecular targets are overexpressed in pre-malignant (adenoma) but not normal colonic mucosa. Fluorescence images collected at WD = 10 mm showed strong intensity from the adenoma (arrow) by comparison with adjacent normal mucosa, Fig. 7b–d (Supplementary Videos 1–3). A signal-to-noise ratio (S/N) of 14.76, 13.04, and 12.49 was measured at λ_ex_ = 488 nm, 660 nm, and 785 nm, respectively. Target-to-background (T/B) ratios of 2.6, 3.1, and 3.9, respectively, were measured. The merged image shows fluorescence from all 3 excitation wavelengths concurrently, Fig. 7e (Supplementary Video 4).

## Discussion

Here, we demonstrate a flexible fiber-coupled wide-field accessory with 2.6 mm diameter and 12 mm length distal tip to collect fluorescence images in multiple spectral bands. These dimensions allow the instrument to pass through the working channel of standard medical endoscopes to collect fluorescence images with high contrast over large mucosal surface areas. A simple, scalable optical design was used to deliver a diffraction-limited collimated beam. Optics were designed to collect fluorescence over a broad spectral bandwidth (λ_ex_ = 500–900 nm) with minimal chromatic dispersion. A miniature scan mirror was placed in the post-objective position to rapidly deflect the beams at maximum total divergence angles of ± 27.4° and ± 22.8° in the X and Y axes, respectively. Multiplexed fluorescence images were collected using excitation at λ_ex_ = 488, 660, and 785 nm. In vivo images were collected from a spontaneous adenoma in the rectum of a live mouse using fluorescently-labeled peptides specific for Prdx1, EGFR, and ErbB2, respectively. T/B ratios of 2.6, 3.1, and 3.9, respectively, were measured. Detection of 3 molecular targets concurrently was demonstrated to address disease heterogeneity. While experimental validation was performed using a pre-clinical model, clinical translation is expected to involve similar peptide concentrations, laser powers, signal levels, and target-to-background ratios.

Standard medical endoscopes are used clinically to rapidly visualize large mucosal surface areas in hollow organs, such as colon. White light illumination is effective to localize pre-malignant lesions that are polypoid in morphology. Pre-malignant lesions that are either flat or subtle in appearance can be identified using fluorescently-labeled ligands that bind to molecular targets specific for disease^18^. A flexible fiber-coupled accessory can be used to collect fluorescence images for use as an adjunct to the conventional white light images. The instrument channel in standard medical endoscopes is typically only a few millimeters in diameter. Also, endoscopes have a large bending angle at the entrance that can obstruct passage of the rigid distal tip^4–6^. A miniature scan mirror placed in the post-objective position can steer a collimated excitation beam with wide angular deflections. Images with a very large FOV can be generated in a miniature instrument package. A side-view geometry allows for the accessory to deliver the excitation beams incident to the mucosal surface with only minimal maneuvering of the distal tip of the medical endoscope. A flexible fiber instrument that can pass forward through this channel may have broad clinical applications for disease detection in hollow organs.

Previously, a multi-modal scanning fiber endoscope (mmSFE) was clinically demonstrated for early detection of esophageal cancer^19,20^. This front-view accessory had rigid tip dimensions of 2.4 mm diameter and 9 mm length. Excitation at λ_ex_ = 638 and 785 nm was delivered concurrently through a single mode optical fiber to collect multiplexed wide-field fluorescence images in 2 channels. A piezo tube actuator was used to generate a spiral scan pattern at a maximum total divergence angle of ~ 70° (± 35°). 6 multi-mode fibers were arranged around the instrument periphery to collect fluorescence. The compact distal tip dimensions allowed for forward passage through the working channel of a standard upper endoscope. Subsequently, instrument dimensions were scaled down for use in the bile ducts to image indeterminant strictures. The rigid tip dimensions were 1.2 mm diameter and 8 mm length with a similar maximum divergence angle of ~ 70°^21^. 2 multi-mode fibers were mounted on either side of the objective to collect fluorescence, and the accessory was passed through the working channel of a standard side-view duodenoscope. By comparison, our accessory has much simpler packaging requirements with comparable performance. The scan mechanism was placed in the post-objective position so that the incident beam was incident along the main optical axis to provide greater alignment tolerance.

Other wide-field endoscope designs have been demonstrated for in vivo imaging. A spectrally-encoded instrument employed a spectral-domain interferometry technique^22^. This endoscope was equipped with a diffraction grating to spectrally diverge a broadband laser beam from a 350 μm diameter single-mode optical fiber on the tissue surface along the X-axis. To form 2D images, a galvanometric mirror was employed to scan in the Y-axis. This system achieved a lateral resolution of 80 μm. Additionally, depth information was acquired using spectral-domain interferometry, resulting in an axial resolution of 135 μm at a depth of 2.8 mm. Using a different approach, a commercial video colonoscope was adapted with a custom illumination module consisting of a halogen lamp and a 635 nm laser diode^23^. A custom imaging head was mounted on the proximal end of the colonoscope, and housed cameras, optical components, and filters. This system was used in human subjects to collect real-time images of white light reflectance and NIR fluorescence from a c-Met peptide concurrently to improve polyp detection during colonoscopy. Furthermore, a side-view optical configuration using a MEMS scanner was previously developed for a confocal endomicroscope^24^.

The imaging performance of wide-field accessory can be improved in several ways. The inner and outer torsional springs can be optimized to increase the deflection angles of the scan mirror. The optical design allows for deflection angles up to ± 19.99° and ± 13.98° in the X and Y axes, respectively, without loss of excitation power. Optical scan angles can reach ± 32.75° and ± 33.40° in the X and Y-axes, respectively, and are comparable with that of the mmSFE. Additional sets of prisms can be introduced around the perimeter of the objective lens to direct the collected fluorescence into multi-mode fibers for increased signal. Also, a more precise alignment of the center-to-center distance between the aspheric and achromatic lenses can improve collimation of the excitation beams and hence image resolution over the range of working distances. The excitation beams can be better collimated to improve image resolution. This can be achieved with a more precise alignment of the center-to-center distance between the aspheric and achromatic lenses and by adjusting the spacing to focus the beam in the middle of the desired range of working distances from 0 to 50 mm. Laser systems and wide-field accessories can be constructed separately for visible and NIR imaging to improve efficiency for light delivery. These systems can be designed to precisely match the core diameters and operating wavelengths of the single-mode fibers. Moreover, additional fluorescence detection channels can be added to further address molecular heterogeneity of mucosal lesions. FITC, Cy5, and IRDye800 were chosen to provide emission spectra that span the full range of visible and NIR wavelengths (λ_em_ = 500–900 nm). The separation between emission peaks is sufficient to add additional fluorophores.

## Methods

### Imaging system

Fiber-coupled solid-state lasers (Coherent #OBIS 488-40 LS FP, TOPTICA #iBEAM-SMART-660-S, and Coherent #OBIS 785-70 LX FP) were used to deliver excitation at λ_ex_ = 488, 660, and 785 nm, respectively. The beams were coupled via FC/APC connectors to a custom wavelength division multiplexer (WDM, Thorlabs, #CDI11086). The combined beams were delivered via a single-mode fiber (SMF_1_, Thorlabs, #630HP) with core/cladding dimensions of 4/125 µm, operating wavelengths of λ = 600–770 nm, and a multi-mode cut-off wavelength of λ_c_ = 570 ± 30 nm. The detachable wide-field accessory contained a separate single-mode fiber (SMF_2_, Thorlabs, #S405-XP) with core/cladding dimensions of 3/125 µm, operating wavelengths of λ = 400–680 nm, and a multi-mode cut-off wavelength at λ_c_ = 380 ± 20 nm terminated with a ferrule (Thorlabs, #30127A3). The excitation beams from SMF_1_ were coupled to the accessory using an FC/APC connector.

Multi-mode fibers (MMF_1_ and MMF_2_, Thorlabs, #FP200ERT) with core/cladding dimensions of 200/225 µm were used to transmit fluorescence, and were terminated with FC/PC connectors. Fluorescence exiting the MMFs was collimated and focused by lenses L_4_-L_6_ (Thorlabs, #F950FC-A) and L_7_ (Thorlabs, #F240FC-780). The beams were directed into 3 channels using a set of dichroic mirrors DM_1_ (IDEX-HS, #FF605-Di02), DM_2_ (IDEX-HS, #FF757-Di01), and DM_3_ (IDEX-HS, #FF925-Di01). DM_1_ transmits and reflects in spectral bands 612–950 and 350–596 nm, respectively. DM_2_ transmits and reflects from 768–1100 and 450–746 nm, respectively. DM_3_ transmits and reflects from 943.5–1600 and 350–906.5 nm, respectively. Long-pass filter LPF_1_ (IDEX-HS, #LP02-488RU-25) transmits from 494–1100 nm, LPF_2_ (IDEX-HS, #LP02-664RU-25) transmits from 673 to 1498 nm, and LPF_3_ (IDEX-HS, #VLP01-785–12.5) transmits from 789 to 1300 nm. Using this scheme, fluorescence was separated into 3 channels for delivery to photomultiplier tube (PMT) detectors PMT_1_ (Hamamatsu, #H7422-40) and PMT_2_-PMT_3_ (Hamamatsu, #H7422-50).

The fluorescence signal was augmented using current amplifiers (Edmund Optics, #59-179), and digitized using a DAQ board (National Instruments, #PCI-6115) at 10 MSa/s. The current amplifier, PMT gain, and laser power were tuned by visually inspecting the images, and these parameters were adjusted until high signal-to-noise (S/N) was achieved. Custom software (National Instruments, LabVIEW 2021) was developed to generate the drive signal to the scan mirror M and to perform real-time data acquisition and image reconstruction.

### Optical design of wide-field accessory

A simple optical design was developed to scale down the dimensions of the fiber-coupled wide-field accessory for forward passage through the instrument channel of a standard medical endoscope. A single mode fiber (SMF_2_) was used to minimize the spot size of the collimated excitation beams. Ray trace simulations were performed using ZEMAX 2013 to identify an aspheric (L_1_, Edmund Optics, #65-273) and achromatic (L_2_, Edmund Optics, #65-564) lens for arrangement in tandem to collimate the excitation beams, and to collect fluorescence over a broad spectral bandwidth (λ_em_ = 500–900 nm) with minimal chromatic dispersion. A miniature scan mirror (M) deflects the beams at 90°. Simulations were performed to determine the image FOV over a wide range of angles. A custom concave lens (L_3_, Tower Optical Corp) was added to provide additional angular divergence for increased image FOV. Fluorescence was collected by L_3_, and descanned by M. Two pairs of right angle triangular prisms (P_1_ and P_2_, Edmund Optics, #66-770) were used to optimize fluorescence collection.

### Scan mirror

A compact 2D scan mirror was designed based on the principle of parametric resonance to perform beam scanning in the X and Y axes^25^. The desired drive signal using this mechanism has a frequency near *2ω*_*0*_*/n* (*ω*_*0*_ is the natural frequency of each resonance mode, n is an integer ≥ 1). Electrostatic comb-drive actuators were used to produce wide angular deflections by tuning the frequency, voltage, and sweep direction of the drive signal. A finite element model (FEM) was developed using COMSOL software to identify the resonance frequencies with desired mode shapes to minimize interference from unwanted parasitic vibrations that can distort the image. These extraneous motions result from mechanical and capacitive coupling of either super or sub-harmonic frequencies near the drive frequency.

The natural frequency of each mode was separated using a spacing of *∆ω* ≥ *0.05ω*. This guideline was based on our previous experience with parametric resonance scanners^26–28^. A pair of inner torsional springs were used to deflect the gimbal frame that supports the reflector at angles larger than that of the comb-drives actuating in the Y-axis. This design maintained a substantial overlap of the comb fingers and accommodates large actuation torques throughout mirror operation even at large angles. The springs were designed to generate the desired resonance frequencies for wide angle scanning while avoiding unwanted, higher-order resonance modes. The electrostatically actuated scan mirror was designed and fabricated using MEMS processes. The device surface, including the reflector, was coated with a ~ 70 nm layer of aluminum using a blanket evaporation process to enhance reflectivity in the visible and NIR spectral regimes^29^.

The frequency response of the scanner in the X and Y axes was measured using a position sensing detector (PSD, On-Track Photonics, #PSM 2-10). The optical window of the accessory was positioned in front of the PSD for testing. Optical scan angles at resonance frequencies were measured by driving each axis separately. The frequency response was evaluated using a down-sweep (high-to-low) and up-sweep (low-to-high) in the drive signal to determine the best direction to achieve resonance. These results were used to select the best set of parameters to produce a large range of angular deflections with minimal parasitic vibrations. The frequencies required to achieve optimal Lissajous scanning in the XY plane at 10 frames per sec (fps) were identified using custom MATLAB software (MathWorks).

### Packaging of wide-field accessory

The rigid distal tip of the accessory was assembled using a stainless-steel outer tube. The scan mirror was packaged in a sealed cavity to mitigate variations in ambient temperature and pressure, and to provide a stable environment. A MEMS holder was 3D-printed using ABS-like plastic (acrylonitrile butadiene styrene) with resolution 63.5 µm (XY) and 203 µm (Z) (Proto Labs, MicroFine resin). The scanner was mounted at a 45° angle, and packaged inside the wide-field accessory in a side-view configuration. Wires (42 AWG, MWS, #B4421241) with 63 µm diameter were looped around electrode anchors to deliver the drive signal. The achromatic lens (L_2_) was placed at the end of the holder first followed by the aspheric lens (L_1_). SMF_2_ was coupled to the accessory using a fiber ferrule (1.0 mm OD, 126 µm ID, 6.4 mm long, Oz Optics, #FER-1.0-126-ZR-6.4-NS), attached to L_1_, and was protected by a polymer tube (Nordson MEDICAL, #Pebax 72D). Two pairs of prisms were glued prior to assembly on the 3D-printed holder. After alignment, one side of the prism was attached directly to the MMF. The position of the optics was fixed using UV glue (Norland, #NOA61). A stainless-steel end tube with dimensions of 2.0 and 1.4 mm OD and ID and 5 mm length, respectively, was inserted inside the proximal side of the outer tube to prevent deformation at the adhesive joint and to isolate the scan mirror from unwanted mechanical vibrations.

### Image characterization

After the scan mirror was packaged in the wide-field accessory, the maximum deflection angles were confirmed by imaging a 2D grid target consisting of black dots with 1 × 1 mm spacing. Fluorophores, including FITC (Sigma-Aldrich, #F6377-100G), Cy5.5 (Lumiprobe, 67020), and IRDye800 (LiCor Biosciences, # 929-80050), were used to stain the target. The lateral resolution of each excitation beam was measured with the scan mirror placed in the neutral position using a beam profiler (Thorlabs, #BP209-VIS). A full-width-at-half-maximum (FWHM) was calculated over a range of working distances 0–50 mm. Lateral resolution was characterized by measuring the FWHM of the PSF in the X and Y axes. The spatial intensity profile of the excitation beam was measured using the beam profiler at WD ranging from 0 to 50 mm. This range spans the typical values used in vivo during endoscopy.

### Ethical approval

All experimental procedures were performed in accordance with relevant guidelines and regulations of the University of Michigan. The mice were housed in pathogen-free conditions and supplied water ad libitum under controlled conditions of humidity (50 ± 10%), light (12/12 h light/dark cycle) and temperature (25 °C) per guidelines of the Unit for Laboratory Animal Medicine (ULAM). In vivo imaging studies were performed with approval of the University of Michigan Committee on the Use and Care of Animals (UCUCA). All methods are reported in accordance with ARRIVE guidelines (https://arriveguidelines.org).

### In vivo imaging

*CPC;Apc* mice were genetically engineered using a Cre recombinase under the control of a Cdx2 promoter (CDX2P-9.5NLS-Cre) and a floxed allele of the *APC* gene^30^. This Cre-regulated somatic mutation in one Apc allele resulted in spontaneous development of adenomas in the distal colon. Anesthesia was induced and maintained via a nose cone with inhaled isoflurane mixed with oxygen at concentrations between 2 and 4% at a flow rate of ∼0.5 L/min. Fluorescently-labeled peptides, including KCC*-FITC, QRH*-Cy5, and KSP*-IRDye800, at concentrations of 1, 10, and 1 mM were mixed and sprayed onto the adenoma and adjacent normal mucosa. These ligands are specific for molecular targets Prdx1, EGFR, and ErbB2, respectively^31–33^. Spectral properties for absorption and emission were measured using a spectrophotometer (Thermo Fisher Scientific, Nanodrop #2000c) and a spectrometer (Ocean Insight, #USB2000 +), respectively. After 10 min for incubation, unbound peptides were washed away using phosphate buffered saline (PBS, Thermo Fisher Scientific, #10,010,023). The wide-field accessory was fixed on a linear stage. The distal tip was positioned at WD = 10 mm. Excitation at λ_ex_ = 488, 660, and 785 nm was delivered concurrently, and wide-field fluorescence images were collected in 3 channels at the same time. This technique mitigated motion artifact from respiratory movements and colon peristalsis.

### Image processing

Wide-field fluorescence images were reconstructed using the trajectory of the scan mirror in a Lissajous pattern^34^. Any phase discrepancies between the drive signal and the scan mirror motion were corrected in real-time using an auto phase correction algorithm^35,36^. The phase was swept in a step size of 0.286° over a range of 2.86° in the X and Y axes to identify the largest variance in neighboring pixel intensities to deblur the image. Grayscale images of fluorescence collected in 3 channels at λ_ex_ = 488, 660, and 785 nm were pseudo-colored in blue, green, and red, respectively, to produce a merged image in real-time. All images were mapped to 400 × 400 pixels and collected at 10 fps. Images and videos were saved in BMP and AVI formats, respectively, and were post-processed using Fiji (ImageJ2) software by adjusting the brightness level and applying a median filter to reduce noise and enhance image quality. The target-to-background (T/B) was calculated as the ratio of mean intensities in the target region versus that in the surrounding boundary of equivalent area. The Otsu and the Markov random field (MRF) algorithms were used to classify pixels as either target or background by maximizing the variance between these two regions^37,38^. Pixel intensities were iteratively updated based on the difference between the intensity and the mean and standard deviation of the two classes. All algorithms were performed using custom MATLAB software (MathWorks).

### Supplementary Information

Supplementary Information.

Supplementary Video 1.

Supplementary Video 2.

Supplementary Video 3.

Supplementary Video 4.

## Data Availability

The datasets used and/or analyzed during the current study are available from the corresponding author on reasonable request.
