# Supplementary material for: Wide-field endoscope accessory for multiplexed fluorescence imaging
Source: Sci Rep. 2023 Nov 9;13:19527. doi: 10.1038/s41598-023-45955-x (PMC10636199; doi:10.1038/s41598-023-45955-x)
Supplement: Supplementary file 1 — Supplementary Information. [file 41598_2023_45955_MOESM1_ESM.docx]

**Wide-field endoscope accessory for multiplexed fluorescence imaging**

Gaoming Li^1+^, Miki Lee^1+^, Tse-Shao Chang^2+^, Joonyoung Yu^2^, Haijun Li^1^, Xiyu Duan^1^, Xiaoli Wu^1^, Sangeeta Jaiswal^1^, Shuo Feng^1^, Kenn R Oldham^2^, Thomas D Wang^1,2,3*^

^1^Division of Gastroenterology, Department of Internal Medicine, University of Michigan, Ann Arbor, MI 48109, USA

^2^Department of Mechanical Engineering, University of Michigan, Ann Arbor, MI 48109, USA

^3^Department of Biomedical Engineering, University of Michigan, Ann Arbor, MI 48109, USA

^+^G. Li, M. Lee, and T-S. Chang contributed equally to this work.

**Corresponding Author:**

^*^Thomas D. Wang, M.D., Ph.D.

Professor of Medicine, Biomedical Engineering, and Mechanical Engineering

H. Marvin Pollard Collegiate Professor of Endoscopy Research

Division of Gastroenterology, University of Michigan

109 Zina Pitcher Pl. BSRB 1522

Ann Arbor, MI 48109-2200

Office: (734) 936-1228

Fax: (734) 647-7950

Email: [thomaswa@umich.edu](mailto:thomaswa@umich.edu)

**Supplementary Figures**


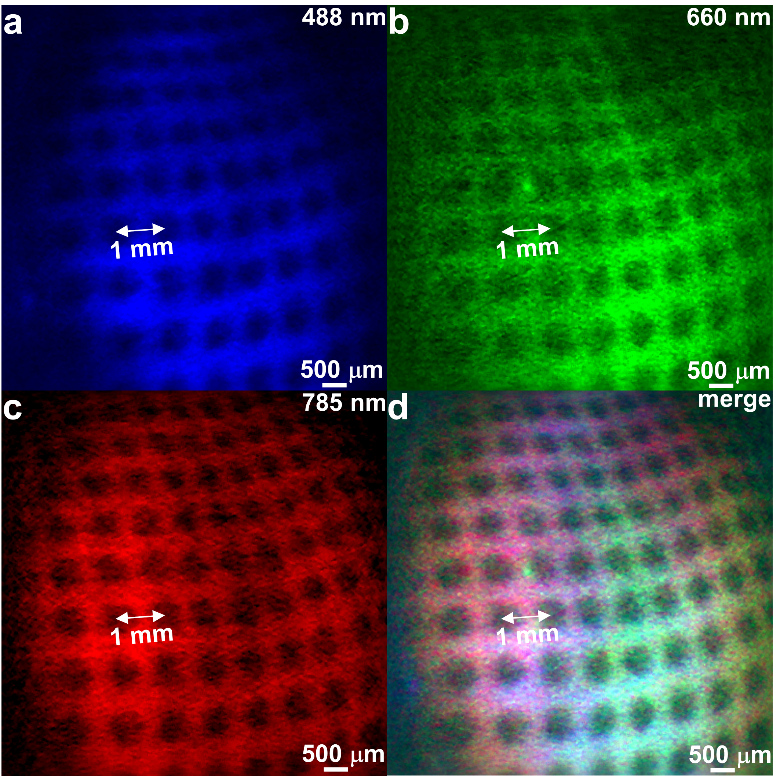


**Fig**. **S1** **–** **Wide field-of-view**. Fluorescence images were collected from a grid target consisting of black dots spaced 1 mm × 1 mm apart at working distance WD = 10 mm. A measured FOV of 10.4 mm × 8.4 mm was supported by detection of 10 and 9 black dots in the X and Y directions, respectively. Excitation at (a) 488, (b) 660, and (c) 785 nm was used after staining the targets with FITC, Cy5.5, and IRDye800, respectively. (d) A merged image was collected using all 3 excitation wavelengths concurrently.


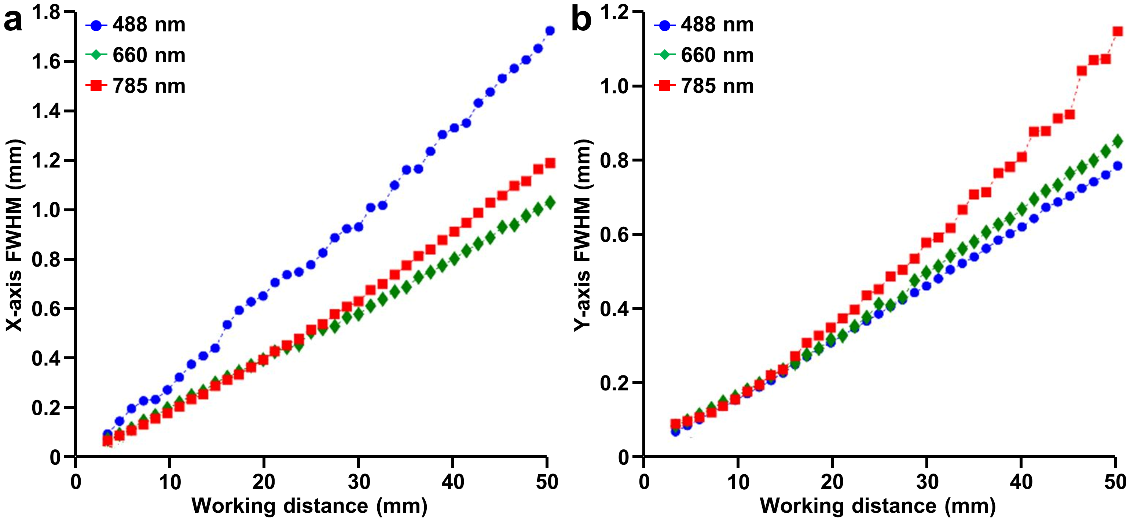


**Fig**. **S2** **–** **Image resolution**. Lateral resolution of the wide-field accessory, defined by the FWHM, was measured in the (a) X and (b) Y axes at WD = 0-50 mm.


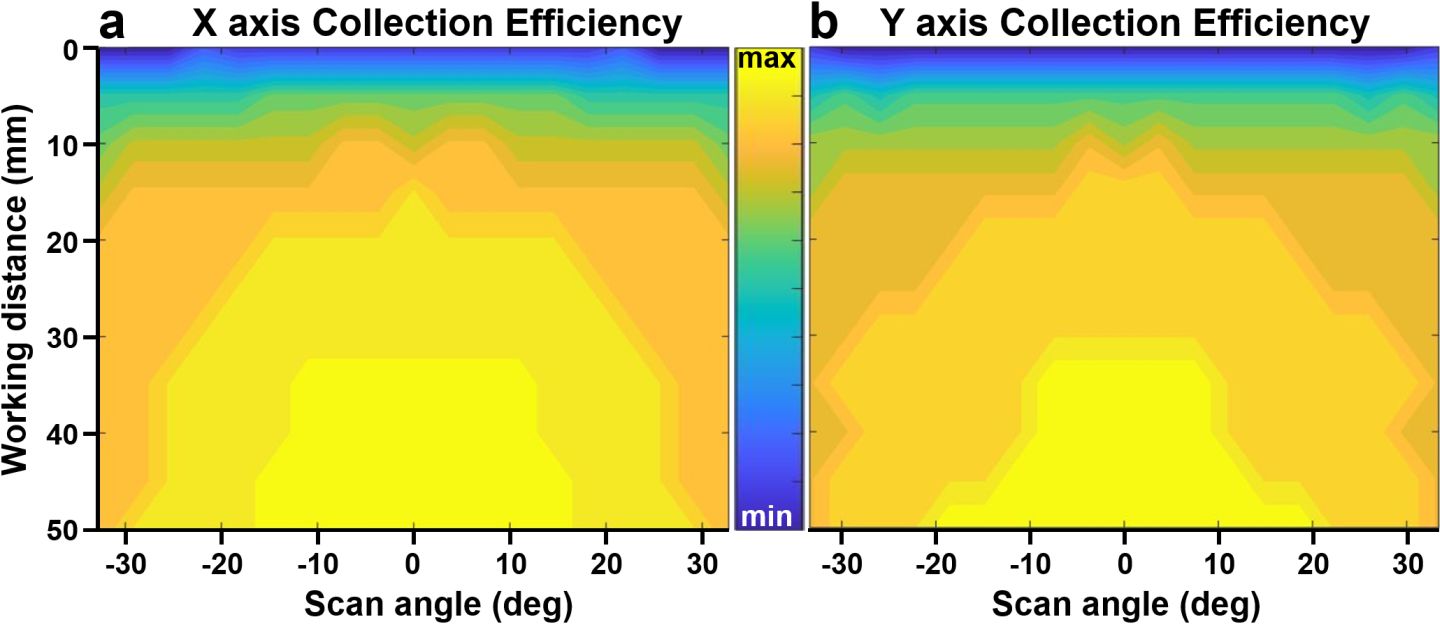


**Fig**. **S3** – **Angular collection efficiency**. Ray trace simulations show the normalized light collection efficiency as a function of scan angle in the (a) X and (b) Y axes.


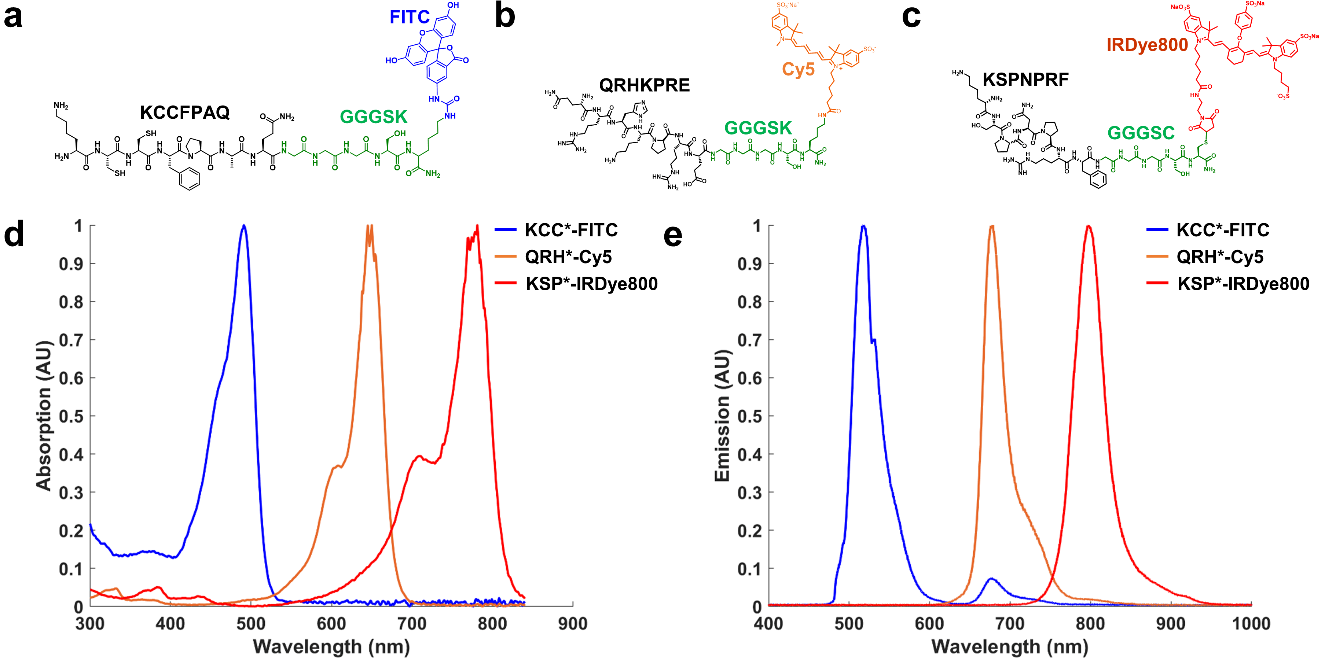


**Fig**. **S4** **–** **Fluorescence spectra**. Biochemical structures for fluorescently-labeled peptides (a) KCC*-FITC (Prdx1), (b) QRH*-Cy5 (EGFR), and (c) KSP*-IRDye800 (ErbB2) are shown. (d) The fluorophores were chosen for use with λ_ex_ = 488, 660, and 785 nm, respectively. (e) Emission spectra show peak fluorescence intensities at λ_em_ = 518, 679, and 798 nm, respectively.

**Supplementary Tables**

| **WD** (mm) | **0** | **5** | **10** | **15** | **20** | **25** | **30** | **40** | **50** |
| --- | --- | --- | --- | --- | --- | --- | --- | --- | --- |
| **FOV_x_** (mm) | 0.82 | 6.04 | 11.25 | 16.47 | 21.69 | 26.90 | 32.12 | 42.55 | 52.98 |
| **FOV_y_** (mm) | 0.69 | 4.91 | 9.12 | 13.33 | 17.55 | 21.76 | 25.98 | 34.41 | 42.84 |

**Table S1** – **Image field-of-view (FOV) versus working distance (WD)**. Ray trace simulations were performed to determine the image FOV_x_ and FOV_y_ in the X and Y axes, respectively, over WD = 0-50 mm.

| **MEMS Scan Angle (X,Y)** (deg) | | **0**  **0** | **10.6**  **0** | **-10.6**  **0** | **0**  **7.3** | **-10.6**  **7.3** | **10.6**  **7.3** | **0**  **-7.3** | **-10.6**  **-7.3** | **10.6**  **-7.3** |
| --- | --- | --- | --- | --- | --- | --- | --- | --- | --- | --- |
| **Aberration** (X,Y) [mr] | **488** (nm) | 0  0 | 0  0 | 0  0 | 0  0 | 0  0 | 0  0 | 0  0 | 0  0 | 0  0 |
|  | **660** (nm) | 0  0 | 0.086  -0.588 | 0.086  0.588 | 0.576  0 | 0.725  0.556 | 0.725  -0.556 | -0.576  0 | -0.607  0.799 | -0.607  -0.799 |
|  | **785** (nm) | 0  0 | 0.118  -0.812 | 0.118  0.812 | 0.795  0 | 1.001  0.767 | 1.001  -0.767 | -0.795  0 | -0.839  1.104 | -0.839  -1.104 |

**Table S2** – **Chromatic aberrations**. Lateral chromatic aberration at different scan angles for wavelengths of 488, 660, and 785 nm were calculated using an optical model.

|  | **FOV_x_ (μm)** | **FOV_y_ (μm)** |
| --- | --- | --- |
| **0%** | 91.54 | 91.54 |
| **50%** | 108.22 | 107.96 |
| **70%** | 123.84 | 123.43 |
| **100%** | 166.78 | 166.65 |

**Table S3** – **Resolution across image FOV**. The image resolution at various positions, including 0, 50, 70, and 100% of the image FOV was determined using an optical model at a working distance of 10 mm.

**Supplementary Videos**

Post-processed fluorescence videos were collected from a colonic adenoma that developed spontaneously in a live *CPC;Apc* mouse. Images were captured at 10 fps. Scale bar: 500 μm.

**Supplementary Video 1** – Video stream collected from prolapsed rectal mucosa demonstrates overexpression of Prdx1 using λ_ex_ = 488 nm following topical administration of KCC*-FITC.

**Supplementary Video 2** – Video stream collected from prolapsed rectal mucosa demonstrates overexpression of EGFR using λ_ex_ = 660 nm following topical administration of QRH*-Cy5.

**Supplementary Video 3** – Video stream collected from prolapsed rectal mucosa demonstrates overexpression of ErbB2 using λ_ex_ = 785 nm following topical administration of KSP*-IRDye800.

**Supplementary Video 4** – Merged video stream collected from prolapsed rectal mucosa demonstrates expression of Prdx1, EGFR, and ErbB2 using λ_ex_ = 488, 660, and 785 nm concurrently following topical administration of KCC*-FITC, QRH*-Cy5, and KSP*-IRDye800.
